# Hyperhomocysteinemia potentiates megakaryocyte differentiation and thrombopoiesis via GH-PI3K-Akt axis

**DOI:** 10.1186/s13045-023-01481-x

**Published:** 2023-07-27

**Authors:** Wenjing Lei, Zhuoliang Liu, Zhiyuan Su, Panpan Meng, Chun Zhou, Xiaomei Chen, Zheng Hu, An Xiao, Miaomiao Zhou, Liping Huang, Yiyue Zhang, Xianhui Qin, Junping Wang, Fengxin Zhu, Jing Nie

**Affiliations:** 1 The State Key Laboratory of Organ Failure Research, National Clinical Research Center of Kidney Disease, Key Laboratory of Organ Failure Research (Ministry of Education), Division of Nephrology, Nanfang Hospital, Southern Medical University, North Guangshou Avenue 1838, Guangzhou, 510515 Guangdong People’s Republic of China; 2 Division of Nephrology, The First Affiliated Hospital of USTC, Division of Life Sciences and Medicine, University of Science and Technology of China, Hefei, 230001 Anhui People’s Republic of China; 3 Division of Cell, Developmental and Integrative Biology, School of Medicine, South China University of Technology, Guangzhou, 510006 Guangdong People’s Republic of China; 4 Department of Obstetrics and Gynecology, Nanfang Hospital, Southern Medical University, Guangzhou, 510515 Guangdong People’s Republic of China; 5 State Key Laboratory of Trauma, Burns and Combined Injury, Chongqing Engineering Research Center for Nanomedicine, Institute of Combined Injury, College of Preventive Medicine, Third Military Medical University, Chongqing, 400038 People’s Republic of China

**Keywords:** Hyperhomocysteinemia, Megakaryocyte, Thrombopoiesis, ScRNA-seq, Growth hormone

## Abstract

Hyperhomocysteinemia (HHcy) is closely associated with thrombotic diseases such as myocardial infarction and stroke. Enhanced platelet activation was observed in animals and humans with HHcy. However, the influence of HHcy on thrombopoiesis remains largely unknown. Here, we reported increased platelet count (PLT) in mice and zebrafish with HHcy. In hypertensive patients (n = 11,189), higher serum level of total Hcy was observed in participants with PLT ≥ 291 × 10^9^/L (full adjusted β, 0.59; 95% CI 0.14, 1.04). We used single-cell RNA sequencing (scRNA-seq) to characterize the impact of Hcy on transcriptome, cellular heterogeneity, and developmental trajectories of megakaryopoiesis from human umbilical cord blood (hUCB) CD34^+^ cells. Together with in vitro and in vivo analysis, we demonstrated that Hcy promoted megakaryocytes (MKs) differentiation via growth hormone (GH)-PI3K-Akt axis. Moreover, the effect of Hcy on thrombopoiesis is independent of thrombopoietin (TPO) because administration of Hcy also led to a significant increase of PLT in homozygous TPO receptor (*Mpl*) mutant mice and zebrafish. Administration of melatonin effectively reversed Hcy-induced thrombopoiesis in mice. ScRNA-seq showed that melatonin abolished Hcy-facilitated MK differentiation and maturation, inhibited the activation of GH-PI3K-Akt signaling. Our work reveals a previously unrecognized role of HHcy in thrombopoiesis and provides new insight into the mechanisms by which HHcy confers an increased thrombotic risk.

*Trial Registration* clinicaltrials.gov Identifier: NCT00794885.

**Supplementary Information:**

The online version contains supplementary material available at 10.1186/s13045-023-01481-x.

To the Editor,

Elevated total serum level of homocysteine (tHcy), known as hyperhomocysteinemia (HHcy) [1], is a risk factor of cardiovascular disease, ischemic stroke, and venous thromboembolism [2, 3]. If untreated, approximately 50% of patients with severe HHcy due to genetic defects suffer from thrombotic events [4, 5], while even moderate HHcy increase the risk of thrombosis [6]. However, the underlying mechanisms remains unclear. Platelets, anucleated cytoplasmic fragments derived from megakaryocyte (MK) [7, 8], are key protagonists in thrombotic disease [9–11]. The aim of the present study is to investigate the impact of HHcy on thrombopoiesis.

Analysis data from 11,189 participants of the China Stroke Primary Prevention Trial [12] revealed that, compared with low PLT (Q1), higher PLT (Q4) was positive correlated with higher tHcy (β = 0.59; 95% CI 0.14,1.04, *p* = 0.010) (Additional file 1: Tables S1–S3 and Fig. S1). Analogous to human, a significant increase in PLT was detected in both male and female mice with HHcy (Fig. 1A; Additional file 1: Fig. S2, S3). Elevated thrombocytes were also observed in a zebrafish transgenic reporter line *Tg(mpl:eGFP)smu4* treated with Hcy (Fig. 1B, C). In a thrombopenia model, the days needed for 50% recovery of PLT from the nadir was shorter in HHcy mice (Fig. 1D). The possible contribution of the spleen to Hcy-increased PLT was excluded since the elevation of PLT remained unaffected in HHcy mice with splenectomy and the half-life of platelet was not affected (Fig. 1E; Additional file 1: Fig. S4). Furthermore, elevation in PLT was observed in *Mpl* mutant mice and zebrafish with HHcy (Fig. 1F–H), suggesting that HHcy facilitates thrombopoiesis independent of TPO.

**Fig. 1:**
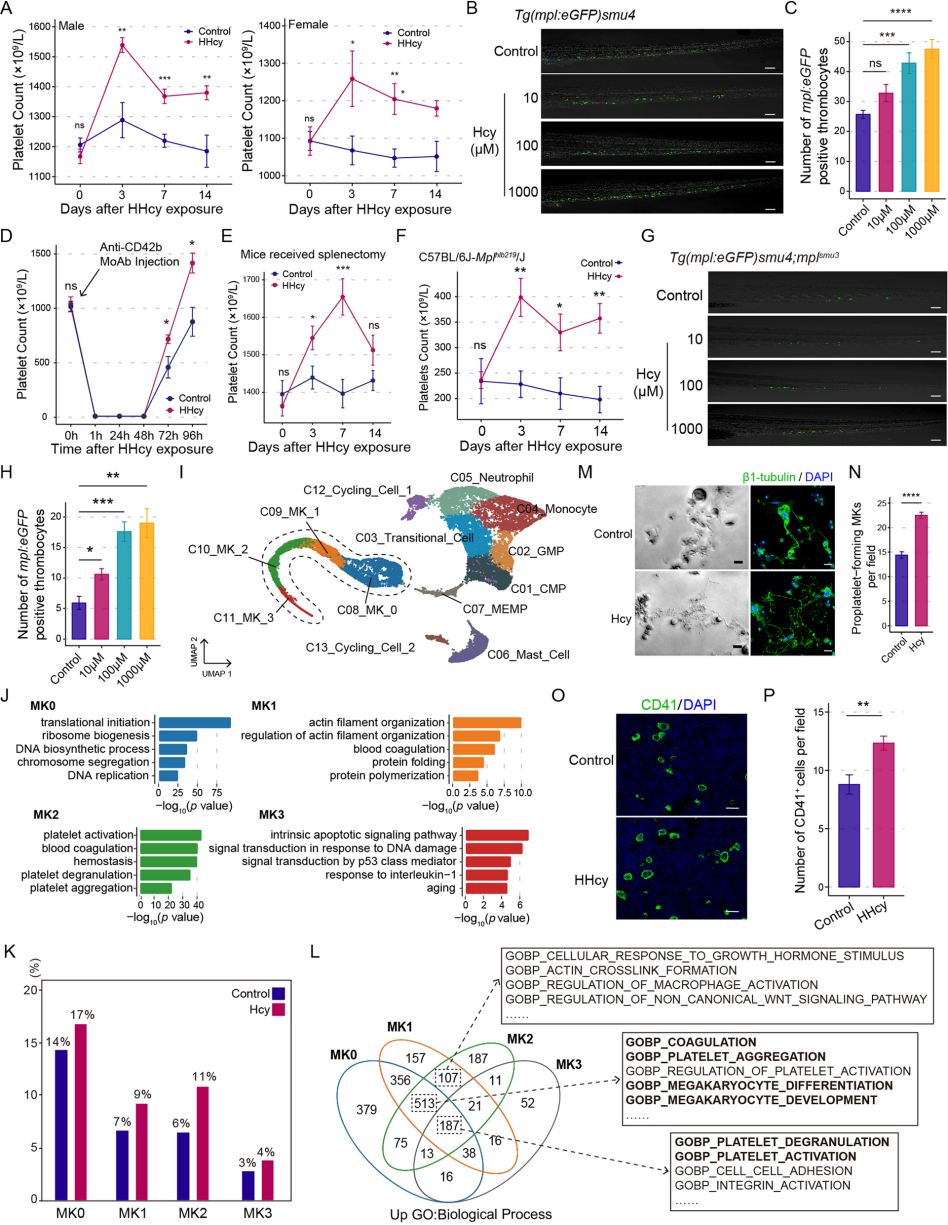
Hcy facilitates MKs differentiation and platelet production. **A** Peripheral PLT in C57BL/6J mice. Significance according to two-tailed unpaired *t* test (n = 8). **B** Representative images for staining and **C** quantification of *mpl*:*eGFP*^+^ cells (green) in zebrafish *Tg(mpl:eGFP)smu4* larvae caudal hematopoietic tissue (CHT) region. Scale bars, 50 μm. Significance according to one-way ANOVA with Tukey multiple comparisons test (n = 10). **D** PLT recovery after platelet depletion by monoclonal rat anti-mouse CD42b antibody (Anti-CD42b MoAb). Significance according to two-tailed unpaired *t* test (n ≥ 5). **E** PLT in splenectomized mice. Significance according to two-tailed unpaired *t* test (n = 8). **F** PLT in C57BL/6J-*Mpl*^*hlb219*^/J mice. Significance according to two-tailed unpaired *t* test (n ≥ 5). **G** Representative images for staining and **H** quantification of *mpl*:*eGFP*^+^ cells (green) at *mpl*-mutational zebrafish *Tg(mpl:eGFP)smu4;mpl*^*smu3*^ larvae CHT region. Scale bars, 50 μm. Significance according to Welch ANOVA test with Dunnett T3 multiple comparisons test (n = 10). **I** 13 cell clusters were displayed by UMAP. Colors indicate cell types. CMP, common myeloid progenitor; GMP, granulocyte-monocyte progenitor; MEMP, megakaryocyte-erythroid-mast cell progenitor; MK, megakaryocyte. **J** Bar diagram showing the representative GO biological process terms of MKs subpopulations. **K** Bar diagram showing the percentage of each MKs subpopulation number to all cells. **L** Venn diagram visualizing the up-regulated biological processes of each MKs subpopulation in Hcy group compared with control group. **M** Representative images of PPF detected by phase contrast imaging (left) and confocal microscopy (right). β1-tubulin (green) and DAPI (blue) were stained; Scale bars, 20 μm. **N** Histogram showing the number of PPF-MKs. Significance according to two-tailed unpaired *t* test (n = 6). **O** Representative images and **P** quantification of CD41^+^ MKs (green) in mice femurs bone marrow by immunofluorescence staining. Scale bars, 50 μm. Significance according to two-tailed unpaired *t* test (n = 8). *p < 0.05, **p < 0.01, ***p < 0.001, ****p < 0.0001, *ns* not significant

To explore the influence of Hcy on the developmental trajectories of MKs, scRNA-seq was performed utilizing cells collected from a hUCB-derived CD34^+^ cell differentiation system. Total 13 clusters including four MK clusters were identified (Fig. 1I; Additional file 1: Fig. S5). Gene ontology (GO) and high-activity regulons (HARs) analysis showed that MK0 represents a less mature population. MK2 is a mature thrombocyte-forming cluster. MK1 serves as a transition state between MK0 and MK2. MK3 highly expresses genes associated with apoptotic signaling (Fig. 1J; Additional file 1: Fig. S6A). Pseudotrajectory analysis revealed a continuous development from MK0 to MK3, which is characterized by a “wave-like” fluctuating gene expression pattern (Additional file 1: Fig. S6B, C). Hcy increased the percentage of MKs, especially MK2 (Fig. 1K). Gene set variation analysis (GSVA) revealed that, compared with control group, biological processes such as megakaryocyte differentiation and megakaryocyte development were significantly up-regulated in MK0-MK2 of Hcy group, indicating Hcy promotes MKs differentiation (Fig. 1L). We further validated the scRNA-seq data. The proplatelet (PPF: β1-tubulin^+^) was enlarged and the amount was increased with Hcy. The number of MKs (CD41^+^) was increased in HHcy mice (Fig. 1M–P). Flow cytometry showed the proportion of MKs (CD41^+^CD42b^+^) was elevated with Hcy (Additional file 1: Fig. S7).

By integrating the scRNA-seq and bulk RNA-seq data from mouse bone marrow MKs (Fig. 2A, B), PI3K-related pathway and response to GH stimulus were the common up-regulated pathways in MKs of Hcy group. Western blotting confirmed Hcy increased the levels of phosphorylated PI3K (p-PI3K) and phosphorylated Akt (p-AKT) (Fig. 2C, D). LY294002, a PI3K inhibitor, blocked Hcy-facilitated MKs differentiation and platelet production (Fig. 2E, F; Additional file 1: Fig. S8). Moreover, when the receptor of GH (GHR) was knocked down in Meg-01 cells by siRNA, Hcy-induced p-PI3K and p-Akt was dramatically attenuated (Additional file 1: Fig. S9). Consistently, no obvious increase in PLT nor the activation of PI3K-Akt signaling was observed in *Ghr*^−/−^ mice with HHcy (Fig. 2G–J). These data indicated that Hcy promotes MK differentiation via boosting the GH-PI3K-Akt axis.

**Fig. 2:**
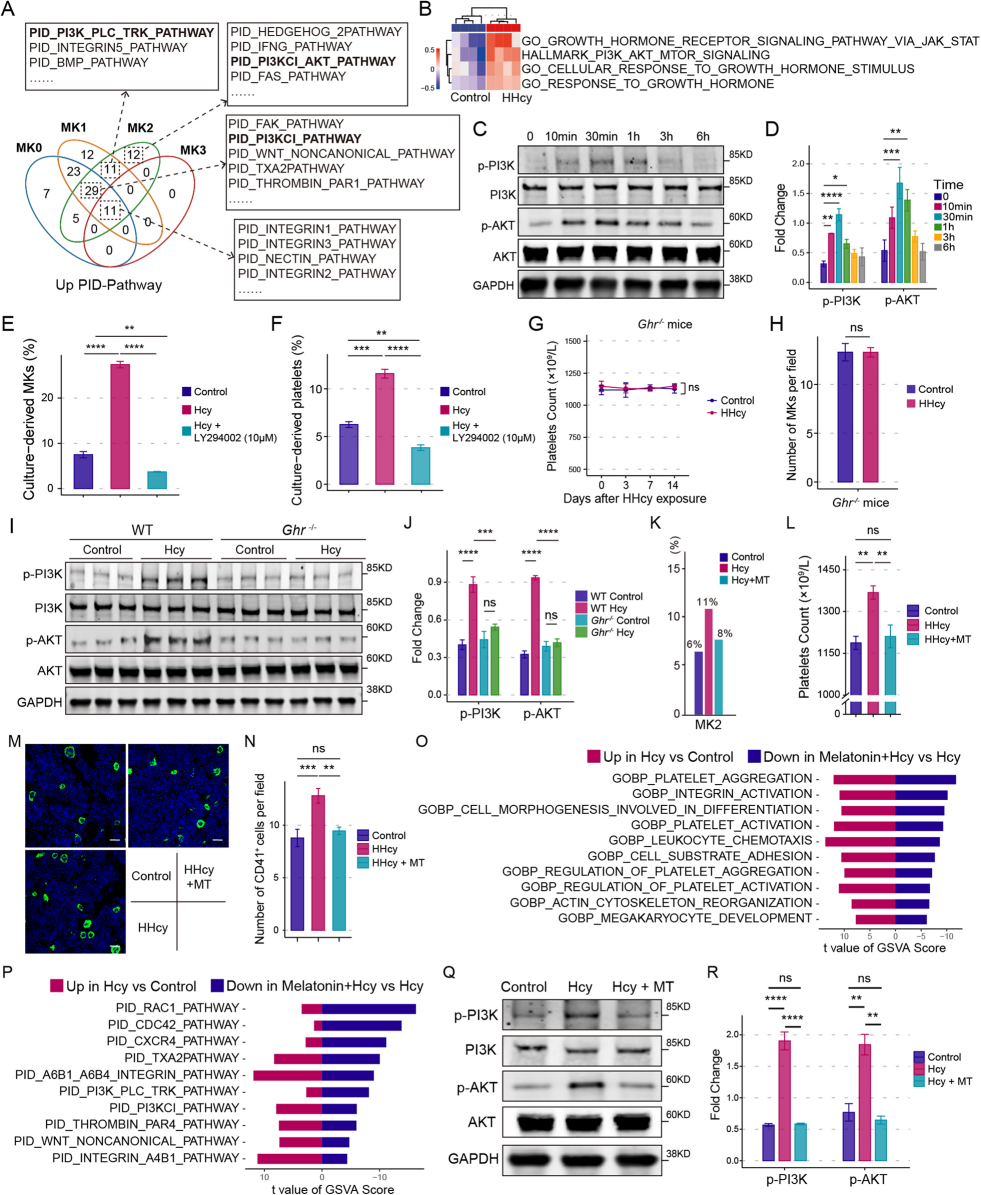
Hcy promotes MKs differentiation via GH-PI3K-Akt axis. **A** Venn diagram visualizing the elevated Pathway Interaction Database pathways of MKs subpopulations in Hcy group. **B** Heatmap showing the relative GSVA scores for each gene set based on bulk RNA-seq of BM MKs (n = 4). **C** and **D** Western blot analysis of p-PI3K and p-AKT (Ser473) in Meg-01 cells after exposure to Hcy (100 μM) for indicated time period. Total PI3K, AKT and GAPDH were used as loading control. Significance according to one-way ANOVA with LSD multiple comparisons test (n = 3). **E** Culture-derived MKs and **F** platelets were analyzed by flow cytometry. Significance according to one-way ANOVA with Tukey multiple comparisons test (n = 3). **G** PLT in *Ghr*^*−/−*^ mice. Significance according to two-tailed unpaired *t* test (n = 6). **H** Quantification of CD41^+^ MKs in femurs bone marrow of *Ghr*^*−/−*^ mice. Significance according to Mann–Whitney test (n = 6). **I** and **J** Western blot analysis of p-PI3K and p-AKT (Ser473) in the bone marrow MKs after exposure to Hcy (100 μM) for 30 min. Significance according to one-way ANOVA with Tukey multiple comparisons test (n = 6). **K** Bar diagram showing the percentage of MK2 to all cells. **L** Peripheral PLT. Significance according to one-way ANOVA with Tukey multiple comparisons test (n = 8). **M** Representative images and **N** quantification of CD41^+^ MKs (green) in femurs bone marrow of mice. Scale bars, 50 μm. Significance according to one-way ANOVA with Tukey multiple comparisons test (n = 8). **O** Bar chart showing the significantly down-regulated GO-BP terms and **P** PID pathways in MKs. *T* values are from the linear model in the limma package. **Q**–**R** Western blot analysis the level of p-PI3K and p-AKT (Ser473) in Meg-01 cells after exposure to Hcy (100 μM) with or without MT (1 μM) for 30 min. Significance according to one-way ANOVA with Tukey multiple comparisons test (n = 3). *p < 0.05, **p < 0.01, ***p < 0.001, ****p < 0.0001, *ns* not significant

Finally, scRNA-seq showed Hcy-increased MK2 proportion was reversed by melatonin (MT) (Fig. 2K). MT blockaded Hcy-facilitated thrombopoiesis in mice without decreasing the level of tHcy (Fig. 2L–N; Additional file 1: Fig. S10A). In addition, Hcy-increased thrombocytes in *Tg(mpl:eGFP)smu4;mpl*^*smu3*^ zebrafish was also reduced by MT (Additional file 1: Fig. S10B, C). As expected, Hcy-elevated MK-associated functions and PI3K-Akt signaling were attenuated by MT (Fig. 2O–R).

Overall, our work demonstrated a role of HHcy in MKs differentiation and characterized the underlying mechanism. Further studies are needed to evaluate the impact of HHcy-promoted thrombocytosis on thrombotic disease.

## Supplementary Information

**Additional file 1**: **Methods**; **Supplemetal Figure** **Legend**; **Table S1.** The association between platelet count and homocysteine; **Table S2.** Characteristics of the study participants by quartailes of platelet count; **Table S3.** Characteristics of the study participants by gender; **Table S4.** Antibody and Reagent used in the study; **Fig. S1.** Flow chart of the study participants in the China Stroke Primary Prevention Trial (CSPPT); **Fig. S2.** Tracking of serum total homocysteine (tHcy) in HHcy mice; **Fig. S3.** Hematological analysis of mice exposed to HHcy treatment for 3 days; **Fig. S4.** HHcy does not affect platelet lifespan; **Fig. S5** Quality control (QC) and cell clusters of scRNA-Seq data; **Fig. S6.** The transcriptome characteristics of four MKs subpopulations; **Fig. S7.** Hcy facilitates MKs differentiation; **Fig. S8.** The strategy of gating platelets; **Fig. S9.** Hcy activates PI3K-Akt axis via GH; **Fig. S10.** Melatonin blockades Hcy-facilitated platelet production.

## Data Availability

The raw data reported in this paper have been deposited in the Genome Sequence Archive (GSA) under the accession number of HRA003377 for scRNA-seq and CRA008832 for mouse MKs bulk RNA-seq, which publicly accessible at https://ngdc.cncb.ac.cn. Individual participant data and other data supporting the findings of this study are available from the corresponding authors on reasonable request, niejing@smu.edu.cn.

## Abbreviations

Hcy: Homocysteine
HHcy: Hyperhomocysteinemia
CSPPT: China stroke primary prevention trial
PLT: Platelet count
ScRNA-seq: Single-cell RNA sequencing
HUCB: Human umbilical cord blood
GHR: Growth hormone receptor
GH: Growth hormone
TPO: Thrombopoietin
rhTPO: Recombinant human TPO
rhSCF: Recombinant human stem cell factor
PI3K: Phosphatidylinositol 3-kinase
AKT: Protein kinase B
CMPs: Common myeloid progenitors
GMPs: Granulocyte-monocyte progenitors
MEMPs: MK-erythroid-mast cell progenitors
MKs: Megakaryocytes
PPF: Proplatelet formation
MT: Melatonin
BM: Bone marrow
DEGs: Differentially expressed genes
GO: Gene ontology
GSVA: Gene set variation analysis
HARs: High-activity regulons
PID: Pathway interaction database
BEAM: Branched expression analysis modeling
PCA: Principal component analysis
UMAP: Uniform manifold approximation and projection
GO-BP: Gene ontology biological process
CHT: Caudal hematopoietic tissue
NC: Scramble siRNA
NHS: N-hydroxysuccinimide
PI: Propidium iodide
DAPI: 4’,6-Diamidino-2-phenylindole dihydrochloride
PRP: Platelet rich plasma
