# Supplementary material for: Hyperhomocysteinemia potentiates megakaryocyte differentiation and thrombopoiesis via GH-PI3K-Akt axis
Source: J Hematol Oncol. 2023 Jul 27;16:84. doi: 10.1186/s13045-023-01481-x (PMC10373258; doi:10.1186/s13045-023-01481-x)

# Hyperhomocysteinemia Potentiates Megakaryocyte Differentiation and Thrombopoiesis via GH-PI3K-Akt axis

**Supplemental Information:**

**Methods**

**Supplemental Figure Legend**

**Supplemental Table 1-4**

**Supplemental Figure 1-10**

## Methods

### Human

The China Stroke Primary Prevention Trial (CSPPT; clinicaltrials.gov identifier: NCT00794885) was a multi-community, randomized, double-blind, controlled trial among 20,702 participants with hypertension but without a history of cardiovascular disease in 32 communities in China. The details and primary results of the CSPPT have been reported[1, 2]. The current analysis included 11,189 participants from the study center in Lianyungang with baseline measures on PLT and Hcy[2]. The flow chart of the participants is presented in Additional file 1: Fig. S1.

### Human data collection and laboratory testing

Baseline data on demographics and lifestyle factors were collected for each participant using a standardized questionnaire. The following factors were included: age, sex, height, weight and smoking status. Body mass index was calculated by dividing weight (kg) by square of standing height (m). Seated blood pressure measurements were obtained by trained research staff after the patients had rested for 10 minutes using a mercury manometer. Triplicate measurements on the same arm were taken, with at least 2 minutes between readings. The mean systolic blood pressure of the 3 independent measures were used in analysis. The measurement of platelet count (PLT) was obtained using a BC-3200 hematology analyzer

(Mindray, Shenzhen, China). Serum total homocysteine (tHcy), fasting glucose, total cholesterol, triglycerides, and creatinine concentrations were measured with automatic clinical analyzers (AU480, Beckman Coulter, Brea, California). All laboratory assays were performed at the core laboratory of the National Clinical Research Centre for Kidney Disease, Nanfang Hospital, Guangzhou, Guangdong, China.

**Animals**

C57BL/6J-$Mpl^{hlb219}$/J mice were obtained from the Jackson Laboratory (Bar Harbor, ME, USA). Mice deficient in *Ghr* ($Ghr^{-/-}$) were purchased from Cyagen Biosciences Inc (Suzhou, Jiangsu, China). C57BL/6J mice were purchased from Beijng Vital River (Beijing, China). All mice in experiments were ranging from 8 to 10 weeks. All procedures performed on the mice were approved by the Southern Medical University and following the Animal Experiments Ethics Committee.

The zebrafish strains Tg(*mpl*:*eGFP*)*smu4* and Tg(*mpl*:*eGFP*)*smu4*; $mpl^{smu3}$ were constructed by Zhang lab[3] and treated under standard conditions[4]. All work involving zebrafish was reviewed by the Animal Ethics Committee or the Animal Research Advisory Committee of South China University of Technology.

To generate a HHcy model, mice were fed with a diet containing 19.56g/kg methionine and water containing 1.8g/L DL-homocysteine

(Sigma; Additional file 1: Table S4) for 14 days.

Melatonin (Sigma, 20mg/kg/day) was injected intraperitoneally at 3:00PM for 7 days. Melatonin was dissolved in ethanol and diluted in sterile water (final concentration of ethanol <4%).

**Platelet depletion**

Platelets were depleted by tail intravenous injection (IV) of 2 μg/g of rat monoclonal antibodies against mouse GPIb (CD42b) R300 (Emfret). Control mice were IV of 2 μg/g control IgG antibody R301(Emfret).

**Platelet life span analysis**

The platelet life span assay and adoptive platelet transfer assay were conducted as described previously[5]. Briefly, mice were intravenously injected with 600 μg NHS-biotin (MedChemExpress). Whole blood was collected at various time points and platelets were isolated and prepared by platelet rich plasma (PRP) preparation kit (Yuanye) and stained with CD41 and streptavidin antibody (Biolegend) in the dark for 30 minutes and flow cytometry was used to analyze platelet life span (FACSCanto II, BD Life Sciences, San Jose, CA, USA).

**Immunofluorescence staining**

Mice exposed to HHcy treatment with or without MT injection for 7 days.

Femurs from mice were fixed in 10% formaldehyde for 24 hours. The prepared samples were embedded in paraffin and sliced into 4-µm-thick sections. For antigen retrieval, slides were microwaved with Tris-EDTA (9.0). Sections were permeabilized with 0.2% Triton X-100 and immunostained with anti-CD41 (abcam) overnight at 4°C then incubated with Goat anti-rabbit Alexa Fluor 488 (ThermoFisher). The nuclei were stained with DAPI (ThermoFisher). The slides were imaged by laser confocal microscope (FV1000, Olympus, Tokyo, Japan). CD41, a surface glycoprotein also known as glycoprotein IIb, has been widely used as a reliable marker MKs. The number of MKs is shown by CD41 positive cells. Ten random fields of view were observed at 200×magnification for each sample and averaged.

**Zebrafish thrombocyte assay**

Zebrafish embryos were soaked into egg water containing indicated drugs for 72 hours in indicated concentration at 36 hours post fertilization and harvested for following assay. Live specimens were anesthetized with tricaine in embryo medium and *Mpl:eGFP*-labeled thrombocytes were counted in Caudal hematopoietic tissue (CHT) with Zeiss microscope (Axio Imager A2, Zeiss, Oberkochen, Germany). For whole-mount confocal images were captured as described previously[3]. Embryos were first stained with goat anti-GFP (Abcam) and then visualized using

donkey anti-goat-Alexa Fluor 488 (Invitrogen) for GFP by Zeiss microscope (LSM800 Zeiss, Oberkochen, Germany).

**Serum tHcy measure**

Blood was collected from anesthetized mice through the retroorbital venous plexus. The serum tHcy was detected by Protable Homocysteine Detector (AUSA 340 II-T, AUSA, Shenzhen, China) with Homocysteine Assay Kit (AUSA).

**Hematological analysis**

Peripheral blood was collected from the tail vein and dissolved in a 1% EDTA solution. Complete blood numbers were counted automatically using a hematology analyzer (BC-6000 Plus, Mindray, Shenzhen, China). Platelets were also counted in accordance with the brecher-chronkite method. Whole blood samples were diluted with 1% ammonium oxalate solution and were then counted in a specific area of the hemocytometer[6].

**Cell culture**

Human umbilical cord blood (hUCB)-derived $CD34^+$ cells were isolated and cultured as previously reported[7]. Briefly, $CD34^+$ cells were purified using an immunomagnetic bead separation system (Stem Cell

Technologies) and cultured in serum-free medium (Stem Cell Technologies) supplemented with 1% penicillin/streptomycin (ThermoFisher) in the presence of 20ng/mL recombinant human stem cell factor (rhSCF; Peprotech) or 20ng/mL recombinant human TPO (rhTPO) for the following analysis. hUCB samples were collected from the Nanfang Hospital, Guangzhou, Guangdong, China. Informed consent was obtained from donors. The study was approved by the hospital's Ethics Committee and Research Ethics Advisory Committee (NFEC-2021-261).

Mouse MKs cells were sorted from bone marrow (BM). Briefly, mouse BM cells were isolated by flushing the femur and tibia then sorted for $CD45^+CD41^+CD61^+$ (Biolegend) propidium iodide$^-$ (PI; Sangon Biotech) cells by flow cytometry (FACSAria Fusion flow cytometer, BD Life Sciences, San Jose, CA, USA). Sorted mouse MKs were cultured in IMDM medium (ThermoFisher) containing 10% fetal bovine serum (ThermoFisher) supplemented with 1% penicillin/streptomycin.

Meg-01 cells were purchased from ATCC (CRL-2021) and maintained in RPMI 1640 medium (ThermoFisher) containing 10% fetal bovine serum supplemented with 1% penicillin/streptomycin. Cells were regularly tested for mycoplasma contamination using LookOut mycoplasma PCR detection kit (Sigma-Aldrich) to ensure mycoplasma-free.

**Measurements of MK differentiation**

rhSCF (20ng/ml) treated hUCB derived $CD34^+$ cells were cultured in the presence of indicated concentration of Hcy and harvested at indicated time. For the differentiation inhibition assay of LY294002 on MKs, $CD34^+$ cells were cultured in the presence of rhSCF (20ng/ml) and Hcy (100μM) with or without LY294002 (10μM) for 10 days. Live cells were obtained using dead cell removal kit (Miltenyi) or PI staining, then labeled with anti-CD41 and anti-CD42b antibody (Biolegend) for 30 minutes at RT in the dark. The cells were fixed and finally analyzed by flow cytometer (FACSCanto II, BD Life Sciences, San Jose, CA, USA).

**Analysis of culture-derived platelets**

Platelets generated from human MKs were assayed as previously described[7]. hUCB derived $CD34^+$ cells were cultured for 10 days with rhTPO (20ng/ml), then treated with Hcy (100μM) with or without pretreated LY294002 (10μM) for another 3 days. The pretreatment with LY294002 was conducted for a period of 30 minutes. All culture contents were harvested and centrifuged at 1000 rpm for 5 minutes. The supernatants were centrifuged at 3000 rpm for 10 minutes. The pellets containing platelets were labeled with CD41, CD42b antibodies and PI

and analyzed by flow cytometer (FACSCanto II, BD Life Sciences, San Jose, CA, USA).

**Proplatelet-forming (PPF) assay**

hUCB derived $CD34^+$ cells were cultured for 10 days with rhTPO (20ng/ml), then treated with Hcy (100μM) for 3 days. The morphology of PPF was captured by phase-contrast images. The expression of β1-tubulin is restricted to the MK/platelet lineage and is indispensable for the cytoskeletal rearrangement in MKs for PPF. Immunofluorescence staining of β1-tubulin is commonly used to mark the amount and morphology of PPF. For confocal microscopy, MKs were fixed with 10% formalin and permeabilized with 0.2% Triton X-100. Cells was stained with anti-β1-tubulin antibody (abcam) and Goat anti-rabbit Alexa Fluor 488 (ThermoFisher). The nuclei were stained with DAPI. The slides were imaged by laser confocal microscope (FV1000, Olympus, Tokyo, Japan). The number of PPF was calculated at a magnification of 200×by observing six random fields for each sample and taking the average.

**siRNA transfections**

Human *GHR* small interfering RNAs (siRNAs) were purchased from Hippobio (Huzhou, Zhejiang, China). A mixture of the following two sense *GHR* siRNA sequences were used: 5′-

GCACCACGCAAUGCAGAUATT-3′, and 5′-GUCAGUUUAACUG GGAUUCAUTT-3′, Scrambled control siRNA was purchased from Ambion. Transient transfection was performed by using Lipofectamine 3000 (Invitrogen) according to the manufacturer's protocols. Human MKs cells were transfected with siRNA at 20 nM concentration for 48 h. The knockdown efficiency of siRNA was detected by Western blot experiments.

**Western blotting**

Cells were harvested and lysed in ice-cold RIPA buffer (Beyotime) containing 1% protease inhibitors (Roche). The extracts were then subjected to sodium dodecyl sulfate (SDS) polyacrylamide gel electrophoresis in MOPS-SDS running buffer and transferred to nitrocellulose filter membrane. After that, the proteins were probed with indicated antibodies. A near-infrared scanning system (Odyssey, Li-COR, Nebraska, USA) was used with fluorescent labeled secondary antibody. The protein expression was analyzed by Image J software.

**Preparation of single-cell suspensions**

rhSCF (20ng/ml) treated hUCB derived $CD34^+$ cells were cultured in the presence of Hcy (100μM) with or without melatonin (MT, 1μM) for 13 days, then cells were collected for 10×Genomics scRNA-seq. Control,

$CD34^+$ cells cultured with rhSCF (20ng/ml); Hcy, $CD34^+$ cells cultured in the presence of rhSCF (20ng/ml) and Hcy (100μM); Hcy +MT, $CD34^+$ cells cultured in the presence of rhSCF (20ng/ml), Hcy (100μM), and melatonin (1μM).

**Single-cell library construction**

For single cell cDNA libraries construction, the cells were analyzed by a 10×Genomics GemCode Single-cell instrument, generating single-cell Gel Bead-In-EMlusion (GEMs). The libraries were generated and sequenced by Chromium Next GEM Single Cell 5' Reagent Kits v3.1 and Illumina NovaSeq 6000 by Genedenovo Biotechnology Co., Ltd (Guangzhou, Guangdong, China) with a paired-end sequencing mode.

**Processing of scRNA-seq data**

Cell Ranger (version 6.0.0) software (https://www.10xgenomics.com/) was used to align and quantify the droplet-based sequencing raw data with default parameters. Specifically, "cellranger count" was used to align the reads to the human reference genome GRCh38 using STAR[8]. The raw output data were processed with Seurat package in R for each individual sample.

**Quality control of scRNA-seq data**

The scRNA-seq data was performed to filter out low-quality and contaminated cells. First, we performed doublet-removal for each dataset using scDblFinder (version 1.6.0) and DoubletDecon (version 1.1.5) with the recommended parameters[9, 10]. Next, for each group sample, cells with mitochondrial, ribosome and hemoglobin genes percentages respectively less than 10%, 50% , and 3% were qualified. In parallel, cells with unique gene counts less than 20000 and detected genes between 300 to 5500 were kept for further analysis. Finally, 25492 cells (Control 8847, Hcy 9798, and Hcy+Melatonin 7047) passed the quality control.

**Dataset integration, cell clustering and dimensionality reduction**

The Seurat (version 4.0.1)[11] package was used to perform downstream analysis and visualization. For merging multiple datasets, we used "SCTransform" to normalize the single-cell RNA-seq datasets, and then "FindIntegrationAnchors" and "IntegrateData" implemented in the Seurat were used to merge samples into one object. "FindIntegrationAnchors" took the union of the top 2000 highly variable genes (HVGs) from each sample. Datasets were scaled using the "ScaleData" function. Principal component analysis (PCA) was conducted using the HVGs, with significant PCs selected based on the elbow of standard deviations. The top 14 significant PCs were selected to perform dimensionality reduction and clustering. Cells were projected into a two-dimension space using

Uniform Manifold Approximation and Projection (UMAP) with default parameters. Cell clusters were identified using the "FindClusters" function from Seurat, based on a Shared Nearest Neighbor (SNN) graph. Finally, 13 clusters ("resolution=0.4") were identified and used for subsequent analysis.

## Identification of differential expression genes and gene ontology enrichment analysis

To annotate the cell clusters, differential expression genes (DEGs) with high discrimination abilities between the groups were identified with the "FindAllMarkers" function in Seurat using the default non-parametric Wilcoxon rank sum test with Bonferroni correction (adjusted p value $< 0.05$ and $log_2$Foldchange $> 0.25$). The cell clusters were annotated based on the DEGs and the well-known cellular markers from the literature. The cluster-specific overrepresented GO biological process (GO-BP) was performed by using ToppGene Suite (https://toppgene.cchmc.org/)[12].

## Trajectory analysis

We applied monocle (version 2.20.0)[13] to determine the lineage differentiation of cell subtypes with potential developmental relationship. The Seurat object was converted to CellDataSet object for importing into

the monocle package. Only genes with the mean expression ≥ 0.1 were used in the trajectory analysis. Then the Discriminative Dimensionality Reduction with Trees (DDRTree) method was used to apply dimensionality reduction. Genes that changed along with the pseudotime were calculated and visualized with the "differentialGeneTest" and "plot_pseudotime_heatmap" meanwhile the genes were clustered into subgroups according to the gene expression patterns. To identify the genes that separate cells into branches, the branch expression analysis modeling (BEAM) analysis were performed and genes resulting from the BEAM analysis and visualized with the "plot_genes_branched_heatmap" function. The enrichment GO terms of the genes in each cluster were calculated with the clusterProfilter package (version 4.0.5)[14].

**Analysis of differential pathway with gene set variation analysis (GSVA)**

Pathway analyses were predominantly performed on the GO-BP and the Pathway Interaction Database (PID) described in the Molecular Signatures Database (MSigDB) v7.2 (https://www.gsea-msigdb.org/gsea/msigdb/index.jsp). Each gene set was pruned to contain unique genes to reduce gene overlaps in each gene set. Next, to score individual cells pathway activity, we applied "gsva" function using standard settings, as implemented in the GSVA package (version 1.40.1)[15]. Then,

significantly different pathways were predicted by a generalized linear model in limma package (version 3.48.3)[16]. Benjamini & Hochberg (BH) adjusted $p$ value < 0.05 was considered statistically significant.

**Identification of high-activity regulons (HARs)**

To identify the transcriptional factors in the cell subpopulation, we used pySCENIC (version 0.11.0) for gene regulatory network analysis[17, 18]. In brief, first, we generated co-expression networks via GRNBoost2 and then inferred direct target genes based on those potential targets for which the motif of the corresponding transcription factors is significantly enriched. Each regulon is then defined as a TF and its direct target genes. The regulon activity score (RAS) in each single cell is calculated through the area under the recovery curve[19]. Subsequently, the Wilcoxon rank sum test was used to analyze the differentially activated regulons among the different cell types. Regulons with an adjusted $p$ value < 0.05 and $log_2$Foldchange > 0.1 were considered to be high-activity regulons (HARs).

**Bulk RNA-seq library preparation and sequencing**

Mice exposed to Hcy treatment for 7 days. Bulk RNA-Seq was performed on mouse bone marrow derived $CD45^+CD41^+CD61^+PI^-$ cells from control and HHcy mice. Library preparation was performed by Gene Denovo

Biotechnology Co (Guangzhou, Guangdong, China). More specifically, total RNA was extracted using Trizol reagent (Vazyme) according to the manufacturer's protocol and enriched by Oligo (dT) beads, while prokaryotic mRNA was enriched by removing rRNA by Ribo-ZeroTM Magnetic Kit (Epicentre). Then the enriched mRNA was fragmented into short fragments using fragmentation buffer and reverse transcripted into cDNA with random primers. Second-strand cDNA were synthesized by DNA polymerase I, RNase H, dNTP and buffer. Then the cDNA fragments were purified with QiaQuick PCR extraction kit (Qiagen), end repaired, dA-Tailing added and ligated to Illumina sequencing adapters. The ligation products were size selected by agarose gel electrophoresis, PCR amplified. All cDNA libraries sequenced on Illumina HiSeq 2500.

**Bulk RNA-Seq data analysis**

RNA-seq raw data were processed and the clean reads were obtained by removing adapter sequences and low-quality reads from raw data using fastp (version 0.12.4)[20]. HISAT2 (version 2.2.1) was used to align trimmed reads to the mm10 mouse reference genome[21]. Uniquely mapped reads were counted using the featureCounts to quantify gene expression[22]. For GSVA, pathway analyses were predominantly performed on the hallmark pathways and GO terms described in the molecular signature database, exported using the msigdbr (version 7.5.1).

Next, to assign pathway activity estimates to individual samples, we applied "gsva" function using standard settings, as implemented in GSVA package (version 1.40.1). To assess differential activities of pathways between control and HHcy groups, we contrasted the activity scores for each sample using a generalized linear model in limma (version 3.48.3) package.

**Statistical analysis**

For human subjects, statistical analysis was performed using R. Participants characteristics are presented as mean±SD or proportions for continuous or categorical variables, respectively. The differences in characteristics according to PLT quartiles were compared using ANOVA tests or chi-squared tests. The associations between PLT and tHcy were estimated using multivariate linear regression models. We conducted multivariate linear regression analyses to assess the association between tHcy and PLT with adjustments for several major covariates. PLT was treated as the independent variable and categorized into four quartile subgroups, with the lowest quartile (Q1) serving as the reference category for comparison. The β coefficient and corresponding 95% confidence interval (CI) represented the association between tHcy and PLT in each subgroup. The analysis was adjusted for covariates including age, sex, body mass index, smoking status, fasting glucose, total cholesterol,

triglycerides, creatinine, and systolic blood pressure. For other study, data are expressed as mean±SEM unless otherwise stated. Statistical analysis was performed using GraphPad Prism 9.4.1. $p<0.05$ was statistically significant.

## References:

1. Huo Y, Li J, Qin X, Huang Y, Wang X, Gottesman RF, Tang G, Wang B, Chen D, He M, Fu J, Cai Y, Shi X, Zhang Y, Cui Y, Sun N, Li X, Cheng X, Wang J, Yang X, Yang T, Xiao C, Zhao G, Dong Q, Zhu D, Wang X, Ge J, Zhao L, Hu D, Liu L, Hou FF. Efficacy of folic acid therapy in primary prevention of stroke among adults with hypertension in China: the CSPPT randomized clinical trial. JAMA 2015;313:1325-1335.

2. Kong X, Huang X, Zhao M, Xu B, Xu R, Song Y, Yu Y, Yang W, Zhang J, Liu L, Zhang Y, Tang G, Wang B, Hou FF, Li P, Cheng X, Zhao S, Wang X, Qin X, Li J, Huo Y. Platelet Count Affects Efficacy of Folic Acid in Preventing First Stroke. J AM COLL CARDIOL 2018;71:2136-2146.

3. Lin Q, Zhang Y, Zhou R, Zheng Y, Zhao L, Huang M, Zhang X, Leung A, Zhang W, Zhang Y. Establishment of a congenital amegakaryocytic thrombocytopenia model and a thrombocyte-specific reporter line in zebrafish. LEUKEMIA 2017;31:1206-1216.

4. Yang L, Wu L, Meng P, Zhang X, Zhao D, Lin Q, Zhang Y. Generation of a thrombopoietin-deficient thrombocytopenia model in zebrafish. J THROMB HAEMOST 2022;20:1900-1909.

5. Zhang W, Ma Q, Siraj S, Ney PA, Liu J, Liao X, Yuan Y, Li W, Liu L, Chen Q. Nix-mediated mitophagy regulates platelet activation and life span. Blood Adv 2019;3:2342-2354.

6. Fukuda T, Asou E, Nogi K, Goto K. Evaluation of mouse red blood cell and platelet counting with an automated hematology analyzer. J VET MED SCI 2017;79:1707-1711.

7. Xu Y, Wang S, Shen M, Zhang Z, Chen S, Chen F, Chen M, Zeng D, Wang A, Zhao J, Cheng T, Su Y, Wang J. hGH promotes megakaryocyte differentiation and exerts a complementary effect with c-Mpl ligands on thrombopoiesis. BLOOD 2014;123:2250-2260.

8. Dobin A, Davis CA, Schlesinger F, Drenkow J, Zaleski C, Jha S, Batut P, Chaisson M, Gingeras TR. STAR: ultrafast universal RNA-seq aligner. BIOINFORMATICS 2013;29:15-21.

9. Germain PL, Lun A, Garcia MC, Macnair W, Robinson MD. Doublet identification in single-cell sequencing data using scDblFinder. F1000Res 2021;10:979.

10. DePasquale E, Schnell DJ, Van Camp PJ, Valiente-Alandi I, Blaxall BC, Grimes HL, Singh H, Salomonis N. DoubletDecon: Deconvoluting Doublets from Single-Cell RNA-Sequencing Data. CELL REP

2019;29:1718-1727.

11. Butler A, Hoffman P, Smibert P, Papalexi E, Satija R. Integrating single-cell transcriptomic data across different conditions, technologies, and species. NAT BIOTECHNOL 2018;36:411-420.

12. Chen J, Bardes EE, Aronow BJ, Jegga AG. ToppGene Suite for gene list enrichment analysis and candidate gene prioritization. NUCLEIC ACIDS RES 2009;37:W305-W311.

13. Qiu X, Mao Q, Tang Y, Wang L, Chawla R, Pliner HA, Trapnell C. Reversed graph embedding resolves complex single-cell trajectories. NAT METHODS 2017;14:979-982.

14. Yu G, Wang LG, Han Y, He QY. clusterProfiler: an R package for comparing biological themes among gene clusters. OMICS 2012;16:284-287.

15. Hanzelmann S, Castelo R, Guinney J. GSVA: gene set variation analysis for microarray and RNA-seq data. BMC BIOINFORMATICS 2013;14:7.

16. Ritchie ME, Phipson B, Wu D, Hu Y, Law CW, Shi W, Smyth GK. limma powers differential expression analyses for RNA-sequencing and microarray studies. NUCLEIC ACIDS RES 2015;43:e47.

17. Van de Sande B, Flerin C, Davie K, De Waegeneer M, Hulselmans G, Aibar S, Seurinck R, Saelens W, Cannoodt R, Rouchon Q, Verbeiren T, De Maeyer D, Reumers J, Saeys Y, Aerts S. A scalable SCENIC

workflow for single-cell gene regulatory network analysis. NAT PROTOC 2020;15:2247-2276.

18. Aibar S, Gonzalez-Blas CB, Moerman T, Huynh-Thu VA, Imrichova H, Hulselmans G, Rambow F, Marine JC, Geurts P, Aerts J, van den Oord J, Atak ZK, Wouters J, Aerts S. SCENIC: single-cell regulatory network inference and clustering. NAT METHODS 2017;14:1083-1086.

19. Suo S, Zhu Q, Saadatpour A, Fei L, Guo G, Yuan GC. Revealing the Critical Regulators of Cell Identity in the Mouse Cell Atlas. CELL REP 2018;25:1436-1445.

20. Chen S, Zhou Y, Chen Y, Gu J. fastp: an ultra-fast all-in-one FASTQ preprocessor. BIOINFORMATICS 2018;34:i884-i890.

21. Kim D, Paggi JM, Park C, Bennett C, Salzberg SL. Graph-based genome alignment and genotyping with HISAT2 and HISAT-genotype. NAT BIOTECHNOL 2019;37:907-915.

22. Liao Y, Smyth GK, Shi W. featureCounts: an efficient general purpose program for assigning sequence reads to genomic features. BIOINFORMATICS 2014;30:923-930.

## Supplemental Figure Legend

**Fig. S1 Flow chart of the study participants in the China Stroke Primary Prevention Trial (CSPPT).**

(A) The flow chart showing the inclusion and exclusion criteria from CSPPT participants.

**Fig. S2 Tracking of serum total homocysteine(tHcy) in HHcy mice.**

(A) Serum tHcy level in male and female C57BL/6J mice with HHcy. Significance according to Welch ANOVA test with Dunnett T3 multiple comparisons test (n=8).

****$p < 0.0001$, ns, not significant.

**Fig. S3 Hematological analysis of mice exposed to HHcy treatment for 3 days.**

(A) Neutrophil, (B) Red blood cells (RBC), (C) Hemoglobin (HGB), and (D) Hematocrit (HCT). Significance according to two-tailed unpaired *t* test (n=8).

ns, not significant.

**Fig. S4 HHcy does not affect platelet lifespan.**

(A) C57BL/6J mice were intravenously injected with 600μg NHS-biotin and then subjected to Hcy treatment as described in method. Whole blood

was collected at indicated time points and platelets were isolated. Flow cytometry was used to analyze the platelet life span. Significance according to two-tailed unpaired *t* test for normality distribution (ND) and Wilcoxon rank sum test for non-normal distribution (NN) (n=8).
ns, not significant.

**Fig. S5 Quality control (QC) and cell clusters of scRNA-Seq data.**

(A) Violin plot illustrating the distribution of QC metrics in turn, including number of features, unique gene counts and the percentage of mitochondrial (Mito) genes, ribosome (Ribo) genes and hemoglobin (HB) genes in each group (solid line represents the QC threshold). A total of 25,492 cells passed QC.

(B) Heatmap showing the scaled expression patterns of top 30 marker genes in each cell cluster with showing representative marker genes.

(C) Violin plots showing the expression levels of representative marker genes across the 13 clusters. Dotted line box indicates MKs markers.

**Fig. S6 The transcriptome characteristics of four MKs subpopulations.**

(A) Heatmap showing the regulon activity score of top 10 high-activity regulons (HARs) in MKs subpopulations.

(B) Pseudotime trajectory of all MKs was predicted by Monocle 2. Top:

MKs were colored by their assigned pseudotime values. Bottom: pseudotime trajectory tree of MKs with different colors indicating different MKs subpopulations.

(C) Heatmap representation of the differentially expressed genes (DEGs) (in row, $q$ value $<10^{-300}$) along the pseudotime (columns) which were clustered hierarchically into six patterns corresponding four phases (indicated by dashed). Right: Associated GO-BP terms are reported for each pattern. Top: Density plots show the enrichment of MKs subpopulations on the pseudotime.

**Fig. S7 Hcy facilitates MKs differentiation.**

hUBC-derived $CD34^+$ cells were cultured in the presence of rhSCF with or without indicated concentration of Hcy for 7, 10, and 13 days. (A) Representative flow cytometry graphs showing the proportion of $CD41^+CD42b^+$ MKs after exposure to indicated concentration of Hcy for 10 days. (B) Histogram showing the proportions of $CD41^+CD42b^+$ cells for each group. Significance according to one-way ANOVA with Tukey multiple comparisons test (n=3).

$*p < 0.05$, $***p < 0.001$, $****p < 0.0001$.

**Fig. S8 The strategy of gating platelets.**

(A) Events located at a delineated site by platelets from human platelet-

rich plasma and stained negative for PI were entered for subsequent analysis.

**Fig. S9 Hcy activates PI3K-Akt axis via GH.**

(A-B) Meg-01 cells were transfected with scramble siRNA (NC) or siRNA against *GHR* (si*GHR*). Two days after transfection, cells were stimulated with Hcy (100μM) for 30minutes. (A) The level of p-PI3K and p-AKT (Ser473) were analyzed by western blot. Total PI3K, AKT and GAPDH were used as loading control. The silencing efficiency of GHR was shown. (B) Histogram showing the fold change of indicated protein significance according to one-way ANOVA with Tukey multiple comparisons test (n=3).

*$p$ < 0.05, **$p$ < 0.01, ns, not significant.

**Fig. S10 Melatonin blockades Hcy-facilitated platelet production.**

(A) Serum tHcy level in C57BL/6J mice after indicated exposure for 7 days. Data are shown as mean±SEM (n=8). Significance according to Welch ANOVA test with Dunnett T3 multiple comparisons test.

(B) Representative images and (C) quantification of staining of *mpl:eGFP* protein at *mpl*-mutational zebrafish *Tg(mpl:eGFP)smu4;mpl*$^{smu3}$ larvae CHT region after treated with Hcy (100μM) with or without melatonin (MT, 1μM) for 72 hours. Green

indicated *mpl:eGFP*$^+$ cells. Scale bars, 50μm. Significance according to one-way ANOVA with Tukey multiple comparisons test (n=10). ***$p<0.001$, ****$p < 0.0001$, ns, not significant.

## Table S1. The association between platelet count and homocysteine.

| PLT, $10^9$/L | N | tHcy, μM (Mean±SD) | Model 1* | | Model 2& | |
|---|---|---|---|---|---|---|
| | | | β(95%CI) | *P* value | β(95%CI) | *P* value |
| **Total participants** | | | | | | |
| **Quartiles** | | | | | | |
| Q1(<210) | 2742 | 14.9±8.6 | Ref | | Ref | |
| Q2(210-<248) | 2813 | 14.8±9.5 | 0.36 (-0.09,0.81) | 0.112 | 0.31 (-0.13,0.76) | 0.165 |
| Q3(248-<291) | 2796 | 14.6±8.9 | 0.40 (-0.05,0.85) | 0.081 | 0.37 (-0.08,0.81) | 0.109 |
| Q4(≥291) | 2838 | 14.4±8.6 | 0.60 (0.14,1.05) | 0.010# | 0.59 (0.14,1.04) | 0.010# |
| **Males** | | | | | | |
| **Quartiles** | | | | | | |
| Q1(<201) | 1082 | 17.2±10.3 | Ref | | Ref | |
| Q2(201-<237) | 1073 | 17.8±12.5 | 0.64 (-0.35,1.63) | 0.204 | 0.67 (-0.31,1.66) | 0.179 |
| Q3(237<276) | 1085 | 17.8±11.7 | 0.70 (-0.28,1.69) | 0.162 | 0.57 (-0.41,1.56) | 0.254 |
| Q4(≥276) | 1095 | 18.1±12.3 | 1.02 (0.03,2.00) | 0.043# | 0.97 (-0.02,1.96) | 0.054 |
| **Females** | | | | | | |
| **Quartiles** | | | | | | |
| Q1(<217) | 1700 | 12.7±5.2 | Ref | | Ref | |
| Q2(217-<256) | 1705 | 12.8±6.5 | 0.15 (-0.23,0.53) | 0.431 | 0.08 (-0.28,0.45) | 0.658 |
| Q3(256-<299) | 1705 | 12.7±5.6 | 0.05 (-0.33,0.43) | 0.804 | 0.12 (-0.25,0.49) | 0.520 |
| Q4(≥299) | 1744 | 13.0±5.5 | 0.30 (-0.08,0.67) | 0.121 | 0.37 (0.00,0.74) | 0.048# |

*Model 1: Adjusted for age and sex;

&Model 2: Adjusted for age, sex, body mass index, smoking status, fasting glucose, total cholesterol, triglycerides, creatinine, and systolic blood pressure; # $p$ <0.05.

PLT=platelet count; tHcy=serum total homocysteine.

**Table S2. Characteristics of the study participants by quartailes of platelet count[*].**

| Characteristics | Platelet count, $10^9$/L | | | | *p* value |
|---|---|---|---|---|---|
| | <210 | 210-<248 | 248<291 | ≥291 | |
| N | 2742 | 2813 | 2796 | 2838 | |
| Age, yrs | 60.0 (7.5) | 59.3 (7.5) | 59.3 (7.7) | 59.3 (7.6) | 0.002 |
| Male, n (%) | 1323 (48.2) | 1185 (42.1) | 1019 (36.4) | 808 (28.5) | <0.001 |
| Body mass index, kg/m2 | 25.4 (3.5) | 25.6 (3.6) | 25.7 (3.6) | 25.8 (3.7) | <0.001 |
| Systolic blood pressure, mmHg | 168.0 (21.8) | 167.7 (20.5) | 167.8 (20.8) | 167.9 (20.7) | 0.951 |
| Current smoking, n (%) | 664 (24.2) | 643 (22.9) | 627 (22.4) | 516 (18.2) | <0.001 |
| Laboratory assays | | | | | |
| Fasting glucose, mmol/L | 6.0 (1.7) | 6.1 (1.9) | 6.1 (1.8) | 6.0 (1.8) | 0.456 |
| Total cholesterol, mmol/L | 5.6 (1.2) | 5.8 (1.2) | 5.8 (1.2) | 5.6 (1.2) | < 0.001 |
| Triglycerides, mmol/L | 1.6 (0.9) | 1.7 (1.0) | 1.8 (2.1) | 1.8 (1.0) | < 0.001 |
| Creatinine, μmol/L | 67.6 (19.8) | 65.8 (17.5) | 64.1 (21.2) | 62.2 (16.6) | < 0.001 |

*Variables were presented as mean (SD), or n (%).

**Table S3. Characteristics of the study participants by gender*.**

| Characteristics | Total | Male | Female | *P* value |
|---|---|---|---|---|
| N | 11189 | 4335 | 6854 | |
| Age, yrs | 59.5 (7.6) | 60.1 (7.7) | 59.1 (7.5) | < 0.001 |
| Body mass index, kg/m$^2$ | 25.6 (3.6) | 25.0 (3.3) | 26 (3.7) | < 0.001 |
| Systolic blood pressure, mmHg | 167.8 (21.0) | 166.1 (20.8) | 169 (21.0) | < 0.001 |
| Current smoking, n (%) | 2450 (21.9) | 2215 (51.1) | 235 (3.4) | < 0.001 |
| Laboratory assays | | | | |
| Fasting glucose, mmol/L | 6.1 (1.8) | 6.0 (1.6) | 6.1 (1.9) | < 0.001 |
| Total cholesterol, mmol/L | 5.7 (1.2) | 5.5 (1.1) | 5.7 (1.2) | < 0.001 |
| Triglycerides, mmol/L | 1.7 (1.3) | 1.6 (1.1) | 1.8 (1.5) | < 0.001 |
| Creatinine, µmol/L | 64.9 (18.9) | 75.6 (21.6) | 58.1 (13.1) | < 0.001 |
| eGFR, mL/(min*1.73 m^2) | 93.6 (15.9) | 92.4 (16.5) | 94.3 (15.4) | < 0.001 |
| Homocysteine, µmol/L | 14.7 (8.9) | 17.7 (11.7) | 12.8 (5.7) | < 0.001 |
| Vitamin B12, pg/ml | 401.7 (166.2) | 398.1 (168.8) | 404 (164.5) | 0.071 |
| Folate, ng/ml | 7.8 (3.3) | 7.0 (3.0) | 8.3 (3.3) | < 0.001 |
| C677T, N (%) | | | | 0.885 |
| CC | 2593 (23.2) | 1011 (23.3) | 1582 (23.1) | |
| CT | 5595 (50.0) | 2155 (49.7) | 3440 (50.2) | |
| TT | 3001 (26.8) | 1169 (27.0) | 1832 (26.7) | |
| Platelet count, 10^9/L | 256.9 (90.0) | 246.0 (96.5) | 263.8 (85.0) | < 0.001 |

*Variables were presented as mean (SD), or n (%).

**Table S4. Antibody and Reagent used in the study.**

| Antibody and Reagent | Company | Catalog number |
|---|---|---|
| FITC anti-mouse CD41 | Biolegend | Cat#133904,RRID:AB_2129746 |
| FITC anti-human CD41 | Biolegend | Cat#303704,RRID:AB_314374 |
| APC anti-human CD42b | Biolegend | Cat#303912,RRID:AB_2113770 |
| APC anti-mouse/rat CD61 Antibody | Biolegend | Cat#104315,RRID:AB_2561733 |
| APC/Cyanine7 anti-mouse CD45 Antibody | Biolegend | Cat#103115,RRID:AB_312980 |
| PE anti-human CD42b | Biolegend | Cat#303905,RRID:AB_314385 |
| PE Streptavidin | Biolegend | Cat# 410503, RRID:AB_2571914 |
| PE anti-mouse NK-1.1 | Biolegend | Cat#156503,RRID:AB_2783135 |
| PE anti-mouse CD19 | Biolegend | Cat#152407,RRID:AB_2629816 |
| PE anti-mouse Ly-6C | Biolegend | Cat#128007,RRID:AB_1186133 |
| PE anti-mouse CD8a | Biolegend | Cat# 100708, RRID:AB_312747 |
| FITC Rat IgG1, κ Isotype Ctrl | Biolegend | Cat#400405, RRID:AB_326511 |
| FITC Mouse IgG1, κ Isotype Ctrl | Biolegend | Cat#400107,RRID:AB_326429 |
| APC Mouse IgG1, κ Isotype Ctrl (FC) | Biolegend | Cat#400121,RRID:AB_326443 |
| APC Armenian Hamster IgG Isotype Ctrl | Biolegend | Cat#400911,RRID:AB_2905474 |
| APC/Cyanine7 Rat IgG2b, κ Isotype Ctrl | Biolegend | Cat#400623,RRID:AB_326565 |
| PE Mouse IgG1, κ Isotype Ctrl | Biolegend | Cat#400112,RRID:AB_2847829 |
| PE Rat IgG2a, κ Isotype Ctrl | Biolegend | Cat#400508,RRID:AB_326530 |
| PE Rat IgG2c, κ Isotype Ctrl | Biolegend | Cat#400707,RRID:AB_326573 |
| Phospho-Akt (Ser473) (D9E) XP® Rabbit mAb | Cell Signaling technology | Cat#4060,RRID:AB_2315049 |
| Akt (pan) (11E7) Rabbit mAb | Cell Signaling technology | Cat#4685,RRID:AB_2225340 |
| Phospho-PI3 Kinase p85 (Tyr458)/p55 (Tyr199) (E3U1H) Rabbit mAb | Cell Signaling technology | Cat#17366,RRID:AB_2895293 |
| PI3 Kinase p85 (19H8) Rabbit mAb | Cell Signaling technology | Cat#4257,RRID:AB_659889 |
| Anti-Growth hormone receptor | Abcam | Cat#ab65304,RRID:AB_2111287 |
| Anti-CD41 | Abcam | Cat#ab134131,RRID:AB_2732852 |
| Anti-beta I Tubulin | Abcam | Cat#ab179511 |
| Anti-GFP | Abcam | Cat#ab6673,RRID:AB_305643 |
| Human Thrombopoietin R Affinity Purified Polyclonal Ab | R&D | Cat#AF1016,RRID:AB_354537 |
| Anti-GAPDH | Proteintech | Cat#60004-1-Ig,RRID:AB_2107436 |
| Goat anti-Rabbit IgG (H+L) Cross-Adsorbed Secondary Antibody, Alexa Fluor™ 488 | ThermoFisher | Cat#A-11008,RRID:AB_143165 |
| Donkey anti-Goat IgG (H+L) Cross-Adsorbed Secondary Antibody, Alexa Fluor™ 488 | ThermoFisher | Cat#A-11055,RRID:AB_2534102 |
| IRDye 800CW goat anti-rabbit IgG(H+L) | LICOR | Cat#926-32211,RRID:AB_621843 |
| IRDye 680LT goat anti-mouse IgG(H+L) | LICOR | Cat#926-68020,RRID:AB_10706161 |
| Antibodies for Platelet Depletion in Mice | EMFRET | Cat#R300, RRID:AB_2721041 |
| Antibodies for Mouse Platelet Depletion (negative control of R300) | EMFRET | Cat#C301,RRID:AB_2734715 |

| | | |
|---|---|---|
| EasySep™ Human CD34 Positive Selection Kit | STEMCELL TECHNOLOGIES | Cat#18056 |
| StemSpan SFEM Medium | STEMCELL TECHNOLOGIES | Cat#09650 |
| rhSCF | PEPROTECH | Cat#300-07 |
| rhTPO | PEPROTECH | Cat#300-18 |
| LookOut mycoplasma PCR detection kit | Sigma | MP0035 |
| Melatonin | Sigma | M5250 |
| DL-homocysteine | Sigma | H4628 |
| Biotin NHS | MedChemExpress | Cat#35013-72-0 |
| LY294002 | MedChemExpress | Cat#HY-10108 |
| Propidium iodide (PI) | Sangon Biotech | Cat#25535-16-4 |
| ProLong™ Diamond (DAPI) | ThermoFisher | Cat#P36962 |
| Penicillin/streptomycin (100×) | ThermoFisher | Cat#10378016 |
| Lipofectamine 3000 | ThermoFisher | Cat#L3000150 |
| RPMI 1640 | ThermoFisher | Cat#61870127 |
| IMDM | ThermoFisher | Cat#12440053 |
| FBS | ThermoFisher | Cat#10099141C |
| isoflurane | RWD | Cat#970-00026-00 |
| RNA isolater Total RNA Extraction Reagent | Vazyme | Cat#R401-01 |
| RIPA buffer | Beyotime | Cat#P1003J |
| Protease Inhibitors Set | Roche | Cat#11206893001 |
| Homocysteine Assay Kit | AUSA | Cat#BC90 |
| Platelet Rich Plasma (PRP) Preparation kit | Shanghai Yuanye | Cat#R30056 |
| Dead Cell Removal Kit | Miltenyi | Cat#130-090-101 |
| Ribo-ZeroTM Magnetic Kit | Epicentre | Cat#MRZH11124 |
| QiaQuick PCR extraction kit | Qiagen | Cat#28104 |

Supplemental Figure 1

A

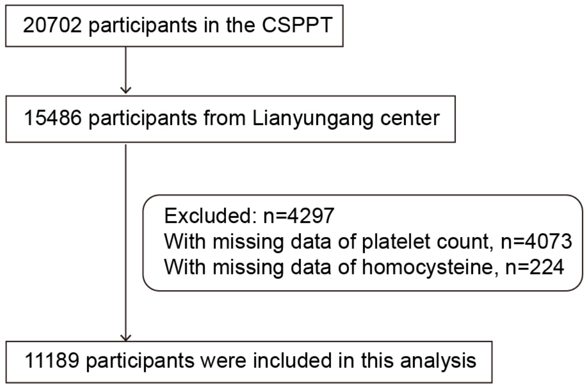

Supplemental Figure 2

A

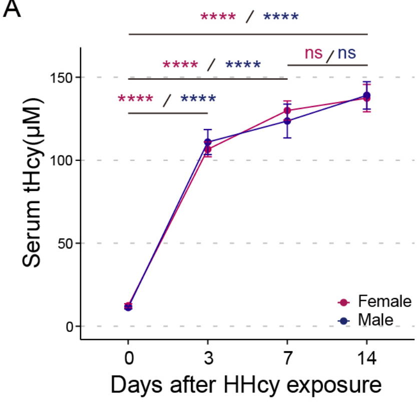

Supplemental Figure 3

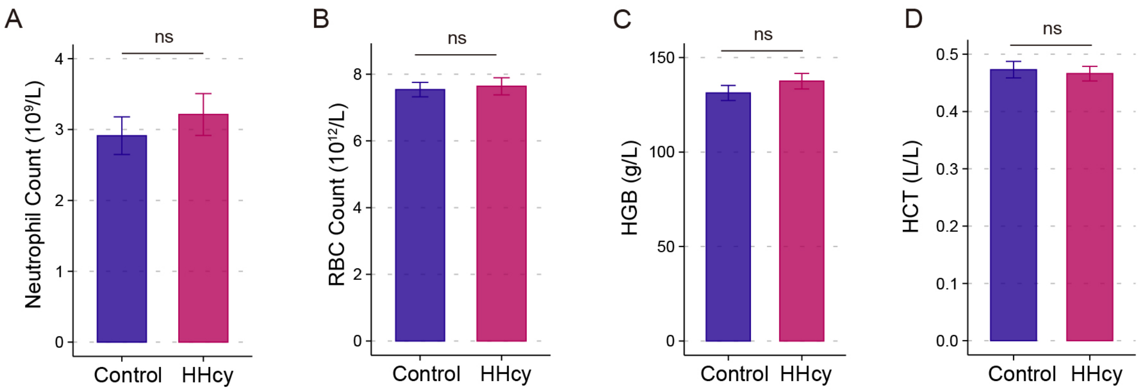

Supplemental Figure 4

A

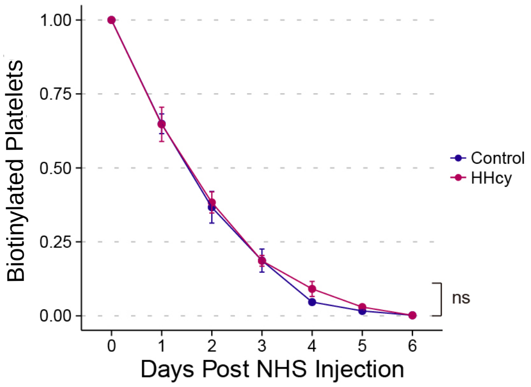

Supplemental Figure 5

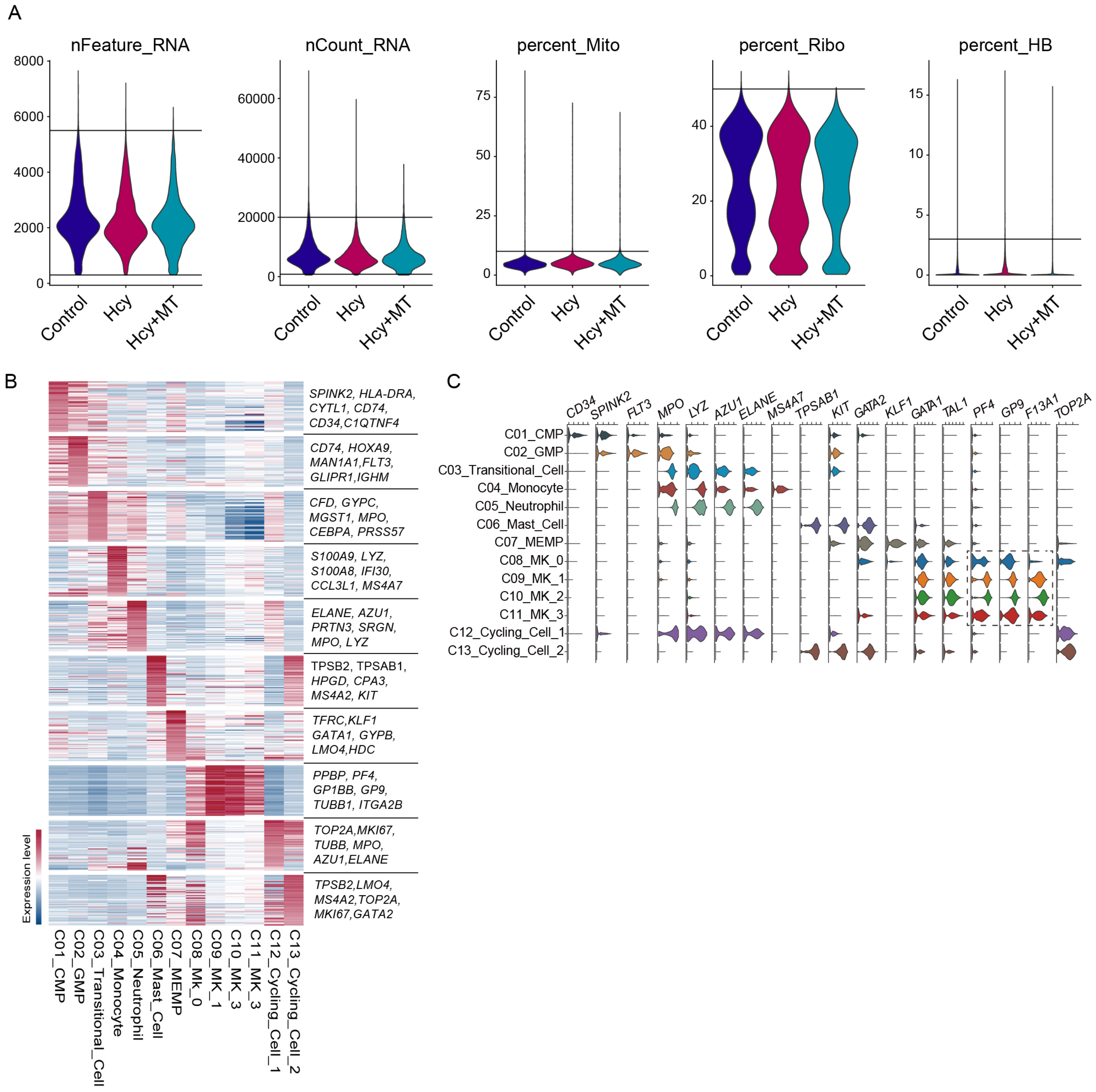

Supplemental Figure 6

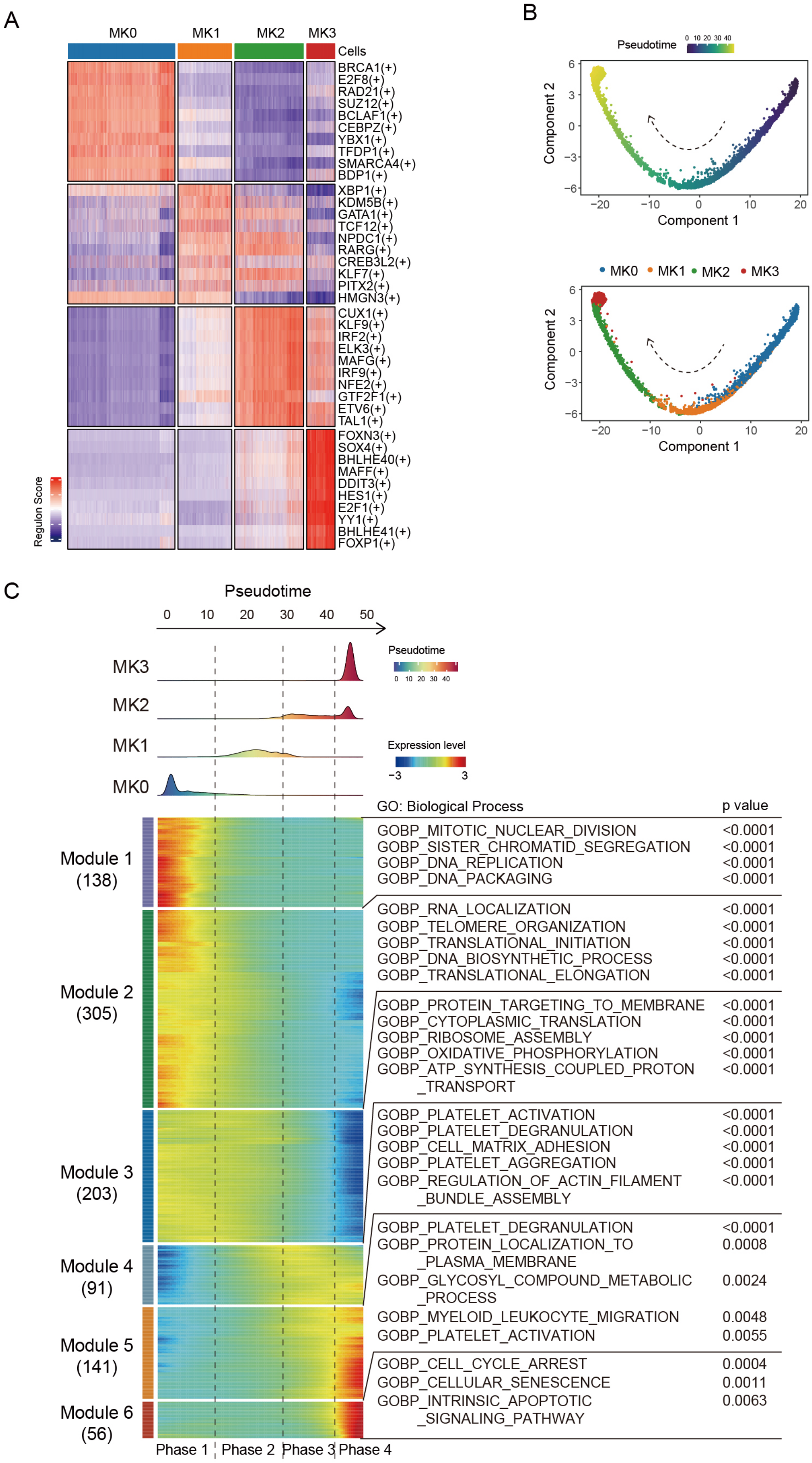

Supplemental Figure 7

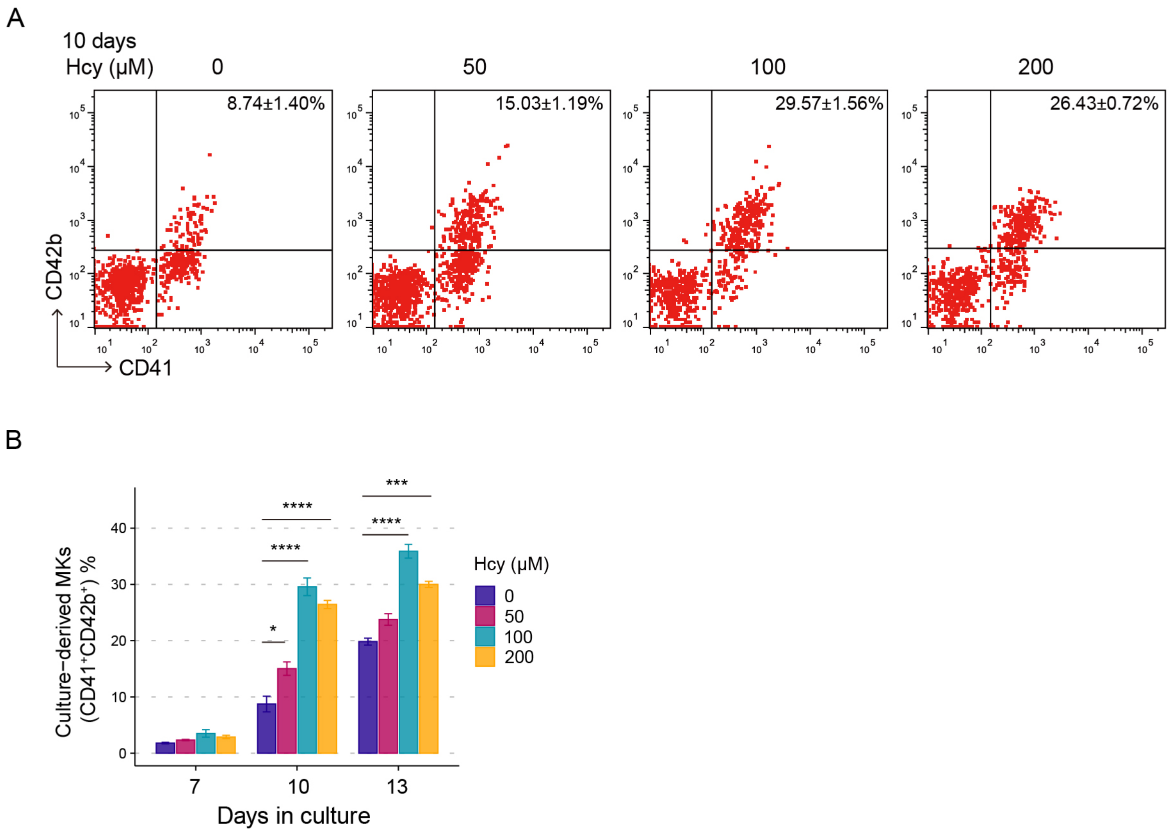

Supplemental Figure 8

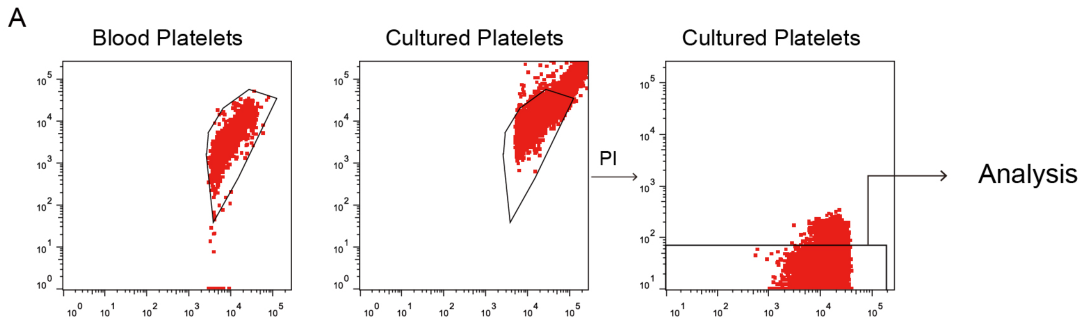

Supplemental Figure 9

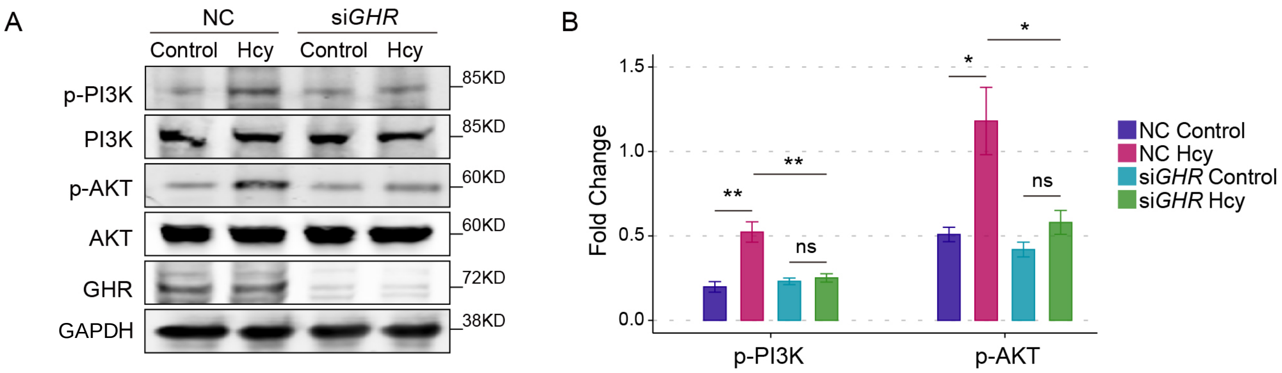

Supplemental Figure 10

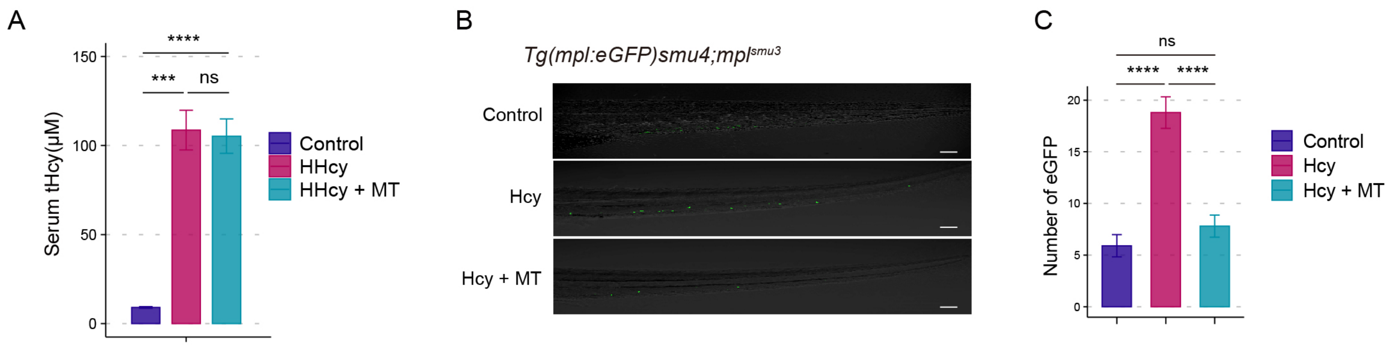

Supplement: Supplementary file 1 — Additional file 1: Methods; Supplemetal Figure Legend; Table S1. The association between platelet count and homocysteine; Table S2. Characteristics of the study participants by quartailes of platelet count; Table S3. Characteristics of the study participants by gender; Table S4. Antibody and Reagent used in the study; Fig. S1. Flow chart of the study participants in the China Stroke Primary Prevention Trial (CSPPT); Fig. S2. Tracking of serum total homocysteine (tHcy) in HHcy mice; Fig. S3. Hematological analysis of mice exposed to HHcy treatment for 3 days; Fig. S4. HHcy does not affect platelet lifespan; Fig. S5 Quality control (QC) and cell clusters of scRNA-Seq data; Fig. S6. The transcriptome characteristics of four MKs subpopulations; Fig. S7. Hcy facilitates MKs differentiation; Fig. S8. The strategy of gating platelets; Fig. S9. Hcy activates PI3K-Akt axis via GH; Fig. S10. Melatonin blockades Hcy-facilitated platelet production. [file 13045_2023_1481_MOESM1_ESM.pdf]
